# Supplementary material for: Noradrenaline causes a spread of association in the hippocampal cognitive map
Source: Nat Commun. 2026 Mar 14;17:3961. doi: 10.1038/s41467-026-70659-x (PMC7618954; doi:10.1038/s41467-026-70659-x)
Supplement: Supplementary file 2 — Reporting Summary [file 41467_2026_70659_MOESM2_ESM.pdf]

nature portfolio

Corresponding author(s): Renée Koolschijn

Last updated by author(s): Jan 29, 2026

Reporting Summary

Nature Portfolio wishes to improve the reproducibility of the work that we publish. This form provides structure for consistency and transparency in reporting. For further information on Nature Portfolio policies, see our Editorial Policies and the Editorial Policy Checklist.

Statistics

For all statistical analyses, confirm that the following items are present in the figure legend, table legend, main text, or Methods section.

n/a	Confirmed	
☐	☒	The exact sample size (*n*) for each experimental group/condition, given as a discrete number and unit of measurement
☐	☒	A statement on whether measurements were taken from distinct samples or whether the same sample was measured repeatedly
☐	☒	The statistical test(s) used AND whether they are one- or two-sided<br>*Only common tests should be described solely by name; describe more complex techniques in the Methods section.*
☐	☒	A description of all covariates tested
☐	☒	A description of any assumptions or corrections, such as tests of normality and adjustment for multiple comparisons
☐	☒	A full description of the statistical parameters including central tendency (e.g. means) or other basic estimates (e.g. regression coefficient) AND variation (e.g. standard deviation) or associated estimates of uncertainty (e.g. confidence intervals)
☐	☒	For null hypothesis testing, the test statistic (e.g. *F*, *t*, *r*) with confidence intervals, effect sizes, degrees of freedom and *P* value noted<br>*Give P values as exact values whenever suitable.*
☒	☐	For Bayesian analysis, information on the choice of priors and Markov chain Monte Carlo settings
☒	☐	For hierarchical and complex designs, identification of the appropriate level for tests and full reporting of outcomes
☐	☒	Estimates of effect sizes (e.g. Cohen's *d*, Pearson's *r*), indicating how they were calculated

Our web collection on statistics for biologists contains articles on many of the points above.

Software and code

Policy information about availability of computer code

Data collection	Matlab v2019b<br>Psychtoolbox V3.0.13<br>3 T Magnetom Prisma (Siemens)<br>EyeLink eyetracker (SR Research)
Data analysis	Matlab v2019b<br>Auryn<br>SPM 12<br>Gannet v3.1.4<br>edf2mat toolbox<br><br>The code used for data analysis is available via the MRC BNDU Data Sharing Platform via https://doi.org/10.60964/BNDU-Z7QY-JP81<br>The code used for the spiking neural network model is available via the MRC BNDU Data Sharing Platform via https://doi.org/10.60964/BNDU-9B3H-A961 and github (https://github.com/p-rakriti/koolschijn_et_al).

For manuscripts utilizing custom algorithms or software that are central to the research but not yet described in published literature, software must be made available to editors and reviewers. We strongly encourage code deposition in a community repository (e.g. GitHub). See the Nature Portfolio guidelines for submitting code & software for further information.

Data

Policy information about availability of data

All manuscripts must include a data availability statement. This statement should provide the following information, where applicable:

- Accession codes, unique identifiers, or web links for publicly available datasets
- A description of any restrictions on data availability
- For clinical datasets or third party data, please ensure that the statement adheres to our policy

All data generated and analysed during this study are included in the manuscript and supporting files. Upon publication the group-level data will be available from the MRC BNDU Data Sharing Platform via https://doi.org/10.60964/BNDU-Z7QY-JP81.

The following dataset was generated:
- fMRI data
- MRS data
- Pupillometry data
- Behavioural data

Research involving human participants, their data, or biological material

Policy information about studies with human participants or human data. See also policy information about sex, gender (identity/presentation), and sexual orientation and race, ethnicity and racism.

Reporting on sex and gender	Participants were stratified according to sex.
Reporting on race, ethnicity, or other socially relevant groupings	Not applicable. We did not collect any data on race or ethnicity as it was not applicable to this research.
Population characteristics	n=44 healthy volunteers. Participants were randomly assigned to one of two groups which were stratified by sex: one group who received a single dose of 10mg atomoxetine (ATX group, n=22, mean age: 23.9 +/- 5.23 yrs, 11 women) and one group who received placebo (PLC group, n=22, mean age: 25.2 +/- 4.80 yrs, 11 women).
Recruitment	Participants were recruited using advertisements on University websites and mailing lists, posters in University departments and word-to-mouth.
Ethics oversight	The Ethics committee of the University of Oxford

Note that full information on the approval of the study protocol must also be provided in the manuscript.

Field-specific reporting

Please select the one below that is the best fit for your research. If you are not sure, read the appropriate sections before making your selection.

☒ Life sciences ☐ Behavioural & social sciences ☐ Ecological, evolutionary & environmental sciences

For a reference copy of the document with all sections, see nature.com/documents/nr-reporting-summary-flat.pdf

Life sciences study design

All studies must disclose on these points even when the disclosure is negative.

Sample size	N=44; this sample size is within the standard range in the field.
Data exclusions	MRS: Reliable model fits were achieved for 38 out of 41 V1 acquisitions (ATX: 20, PLC: 18) and for 37 out of 43 LOC acquisitions (ATX: 19, PLC: 18).<br>fMRI: one participant was excluded due to excessive motion.<br>Pupillometry: data from 34 out of 44 participants was included (ATX: 15, PLC: 19).<br>Behaviour, topological distance store: Topological distance score, with a lower score for the ATX group suggesting evidence for overgeneralisation data from participants who correctly remembered the entire cognitive map was excluded, including 37 out of 44 participants (ATX: 21, PLC: 16)
Replication	N/A
Randomization	Participants were randomly allocated to a treatment group (drug or placebo).
Blinding	Double-blinded.

Reporting for specific materials, systems and methods

We require information from authors about some types of materials, experimental systems and methods used in many studies. Here, indicate whether each material, system or method listed is relevant to your study. If you are not sure if a list item applies to your research, read the appropriate section before selecting a response.

Materials & experimental systems

n/a	Involved in the study
☒	☐ Antibodies
☒	☐ Eukaryotic cell lines
☒	☐ Palaeontology and archaeology
☒	☐ Animals and other organisms
☒	☐ Clinical data
☒	☐ Dual use research of concern
☒	☐ Plants

Methods

n/a	Involved in the study
☒	☐ ChIP-seq
☒	☐ Flow cytometry
☐	☒ MRI-based neuroimaging

Plants

Seed stocks	*Report on the source of all seed stocks or other plant material used. If applicable, state the seed stock centre and catalogue number. If plant specimens were collected from the field, describe the collection location, date and sampling procedures.*
Novel plant genotypes	*Describe the methods by which all novel plant genotypes were produced. This includes those generated by transgenic approaches, gene editing, chemical/radiation-based mutagenesis and hybridization. For transgenic lines, describe the transformation method, the number of independent lines analyzed and the generation upon which experiments were performed. For gene-edited lines, describe the editor used, the endogenous sequence targeted for editing, the targeting guide RNA sequence (if applicable) and how the editor was applied.*
Authentication	*Describe any authentication procedures for each seed stock used or novel genotype generated. Describe any experiments used to assess the effect of a mutation and, where applicable, how potential secondary effects (e.g. second site T-DNA insertions, mosiacism, off-target gene editing) were examined.*

Magnetic resonance imaging

Experimental design

Design type	Task-based, event-related.
Design specifications	4 scan blocks (2 analyzed here, 133 trials each). On each trial, stimuli were presented for 1000 ms. The inter-trial interval was selected from a truncated gamma distribution with a mean of 2.3 s, minimum of 1.3 s and maximum of 14 s.
Behavioral performance measures	Button presses were recorded as part of a cover task. Participants were instructed to press a button on an MR compatible button box using their right index finger when they identified "oddball" stimuli, but not if the stimulus was familiar.

Acquisition

Imaging type(s)	structural, functional, MR spetroscopy
Field strength	3 Tesla
Sequence & imaging parameters	functional: multiband echo-planar imaging (EPI) sequence, with multiband factor 3, TR = 1.235 s, TE = 20 ms, flip angle = 65 degrees, field of view = 216 mm, and a voxel resolution of 2×2×2 mm3. All scans were of axial orientation angled to the long-axis of the hippocampus, covering the whole brain.<br>structural: 192 1 mm axial slices, in-plane resolution of 1x1 mm2, TR= 1.9 s, TE = 3.97 ms, and field of view = 192 mm<br>field map: TE1 = 4.92 ms, TE2 = 7.38 ms, whole-brain coverage, voxel size 2x2x2 mm3.<br>MRS: locally developed version of the CMRR Spectroscopy Package MEscher-GArwood Point RESolved Spectroscopy (MEGA-PRESS) sequence in two 2 x 2 x 2 cm3 VOIs, with TE = 68 ms, TR = 1.5 s, flip angle 90 deg, ON editing pulse 1.9 ppm, OFF editing pulse 7.5 ppm.
Area of acquisition	structural and functional: whole brain<br>MRS: V1 and LOC

Diffusion MRI ☐ Used ☒ Not used

Preprocessing

Preprocessing software	SPM 12
Normalization	Linear transformation from functional to structural, non-linear transformation from structural to standard MNI space.
Normalization template	Group standardized space (MNI152).
Noise and artifact removal	Bias field correction, distortion correction using field map, motion correction using 6 scan-to-scan motion parameters produced during realignment.
Volume censoring	N/A

Statistical modeling & inference

Model type and settings

3 first level designs:
GLM 1 (repetition suppression): univariate with 14 explanatory variables and 6 motion parameters per scan block.
GLM 2 (11-node XSS): univariate with 14 explanatory variables and 6 motion parameters per scan block.
GLM 3 (6-node XSS): univariate with 17 explanatory variables and 6 motion parameters per scan block.

7 second level designs:
For GLM 1 (1 model): random effects model without any covariates.
For GLM 2 (5 models): random effects model without any covariates, and with GABA+ in LOC, pupil response and behaviour (overgeneralisation errors and mean rank proximity) as covariates.
For GLM 3 (1 model): random effects model without any covariates.

Effect(s) tested

GLM 1 (main effect): parametric regressor of n trials since last stimulus presentation contrasted against baseline.
GLM 2 (main effect): parametric regressor of link distance contrasted against baseline.
GLM 2 (correlation GABA+): covariance between parametric regressor and GABA+ in LOC
GLM 2 (correlation pupil response): covariance between parametric regressor and the pupil dilation effect in response to a surprising stimulus
GLM 2 (correlation behaviour 1): covariance between parametric regressor and overgeneralisation errors
GLM 2 (correlation behaviour 2): covariance between parametric regressor and mean rank proximity
GLM 3 (main effect): mean of 2 parametric regressors (link distance previous trial (t-1) and link distance trial before previous (t-2)) contrasted against baseline.

Specify type of analysis: ☐ Whole brain ☐ ROI-based ☒ Both

Anatomical location(s): ROI in LOC centred on MRS voxel, anatomical ROIs of hippocampus and parahippocampus.

Statistic type for inference (See Eklund et al. 2016)	cluster-wise, with the significance level defined as p<0.05
Correction	p<0.01 uncorrected before using whole-brain FWE

Models & analysis

n/a	Involved in the study
☒	☐ Functional and/or effective connectivity
☒	☐ Graph analysis
☒	☐ Multivariate modeling or predictive analysis
