# Supplementary material for: Noradrenaline causes a spread of association in the hippocampal cognitive map
Source: Nat Commun. Author manuscript; Available in PMC 2026 Apr 1. (PMC7618954; doi:10.1038/s41467-026-70659-x)
Supplement: Supplementary file 1 [file EMS213140-supplement-Supplementary_Information.docx]

**Supplementary Information**

**Supplementary Figures**

 

**Supplementary Figure 1 | Blinding data for participants and experimenters**

To assess the effectiveness of the double-blinding procedure (see also Supplementary Table 1), after completion of the first study day the participants and the experimenter were asked whether they thought they/the participant received drug or placebo. **a** In the atomoxetine group, 41% of participants correctly guessed that they had received atomoxetine. **b** In the placebo group, 68% of participants correctly guessed that they had received placebo. **c** The experimenter correctly guessed for 50% of participants that they had received atomoxetine. **d** The experimenter correctly guessed for 82% of participants that they had received placebo. 

 

**Supplementary Figure 2 | Example trials from learning task**

**a-b** Two example bird pairs situated within their contextual cues. Red circles indicate the location of the bird stimuli. Bird images are by William Norris under a CC0 license. Background images are by Naomi Hébert (a) and Matt Bango (b) under a CC0 license. 

 

**Supplementary Figure 3 | Inference task performed in day 1 and 5**

**a** After the learning task (Fig. 1d), participants inferred indirect relationships between bird stimuli in the absence of feedback, to encourage cohesive map learning (‘*Inference task’*). **b-c** Upper: Bootstrap-coupled estimation (DABEST) plots. Black dot, mean; black ticks, 95% confidence interval; filled curve, sampling error distribution. Lower: memory accuracy (mean +/- SEM). Lower right: null distribution of the group differences generated by permuting subject labels, purple dot: true group difference. **b** There was no significant difference in performance between groups on the inference task (ATX – PLC (n=22:22): permutation test p=0.128). **c** On Day 5 participants repeated the inference task. Again, there was no significant difference in inference task performance between the groups (ATX – PLC (n=22:22): permutation test p=0.124). 

**Supplementary Figure 4 | Ring topology task: elevated noradrenaline during learning does not affect explicit and implicit memory accuracy but does affect estimates of topological distance.** 

**a** Schematic of the Day 5 “*Ring topology task*”, in which participants were asked to piece together all explicit and implicit associations experienced on Day 1, by dragging two bird stimuli to each scene to construct the entire underlying topology. **b-d** Upper: Bootstrap-coupled estimation (DABEST) plots. Black dot, mean; black ticks, 95% confidence interval; filled curve, sampling error distribution. Lower left: memory accuracy (mean +/- SEM). Lower right: grey: null distribution of the group differences generated by permuting subject labels; purple dot: true group difference. **b** Overall accuracy for the explicitly learned bird-bird associations in the *Ring topology task*. No significant difference in explicit memory accuracy was observed between groups (ATX – PLC (n=21:22): permutation test p=0.487). **c** Overall accuracy for the implicit bird-scene associations in the *Ring topology task*. No significant difference in implicit memory accuracy was observed between groups (ATX – PLC (n=22:21): permutation test p=0.259). **d** Overall *‘Topological distance’* on the *Ring topology task*, defined as the average link distance between the bird-bird and bird-scene choices made by each participant. For example, a correct pairing between bird ‘1’ and ‘2’, or between bird ‘1’ and sofa ‘A’ would be given a rank score of 1, while an incorrect pairing between bird ‘1’ and bird ‘3’, or between bird ‘1’ and sofa ‘B’ would be given a rank score of 2, and so on. A significant group difference was observed for the *Topological distance* score, with a lower score for the ATX group suggesting evidence for overgeneralisation constrained by the underlying topology of the task (excluding participants who correctly remembered the entire cognitive map, ATX – PLC (n=21:16): permutation test p=0.027). 

 

**Supplementary Figure 5 | MRS quality measures in LOC showed no significant difference between groups**

**a-b** Lateral Occipital complex (LOC) is a brain region implicated in visual object processing, including basic object recognition, scene perception and processing of object shape^44,45^. **a** ROI consisting of two 10mm radius spheres, where the right sphere was centered on the group-average MRS voxel positioned in LOC (Fig. 3c), and the left sphere being mirrored along the x-axis. Used for small volume correction (SVC) for the bird stimuli repetition suppression contrast. **b** During the scan task, the BOLD response in LOC was significantly modulated by task stimuli, as measured using repetition suppression across all participants (SVC with ROI shown in *A*, n=43, t_42_=4.23, p=0.010, MNI coordinates, Supplementary Table 2). The repetition suppression contrast was generated with a parametric regressor indicating the number of trials since the same stimulus was last presented (see *Methods*). This LOC repetition suppression effect is shown in red-yellow overlapped with the position of MRS voxel in blue (Fig. 3c). In response to ATX, changes in Glx/GABA+ in LOC were therefore expected to result in task-relevant neural changes. **c** The average signal-to-noise ratio (SNR) of N-acetylaspartate (NAA), a reference metabolite, as determined by Gannet (see *Methods*) was for 187.1% (n=19, SEM 10.24) for the ATX group and 183.2% (n=18, SEM 8.39) for the PLC group. There was no significant difference between groups (ATX – PLC: permutation test p=0.388). **c** The average full-width-at-half-max (FWHM) of NAA as determined by Gannet was 8.638 Hz (SEM 0.296) for ATX and 8.777 Hz (SEM 0.240) for PLC. There was no significant difference between groups (ATX – PLC: permutation test p=0.363). **e** The average fit error for Glx as determined by Gannet was 6.820% (SEM 0.835) for ATX and 6.447% (SEM 0.563) for PLC. There was no significant difference between groups (ATX – PLC: permutation test p=0.379). **f** The average fit error for GABA+ as determined by Gannet was 8.814% (SEM 0.632) for ATX and 8.344% (SEM 0.710) for PLC. There was no significant difference between groups (ATX – PLC: permutation test p=0.312).

**Supplementary Figure 6 | Elevating noradrenaline gives no significant change in Glx or GABA+ in V1**

In contrast with the significant group difference reported in LOC (Fig. 3e–f), no significant difference in inhibitory processing was observed in V1. The lack of effect in V1 may be explained by reduced SNR in V1 compared to LOC, which may be attributed to spatial inhomogeneities in the static magnetic field (B0) or higher physiological noise levels in V1^84^. This result could also be attributed to differences in the availability of noradrenaline receptors between LOC and V1^85–89^, or cortically varying effects of atomoxetine on other neuromodulators, like prefrontal dopamine^24,90^ or hippocampal acetylcholine^91,92^. **a** Anatomical location of the 2 x 2 x 2 cm^3^ MRS VOI, positioned in V1. Cumulative map across 38 participants. **b-d** We used MRS to quantify the effect of atomoxetine on excitatory and inhibitory tone in V1, ~3.5 h after drug intake. Upper: DABEST plots. Black dot, mean; black ticks, 95% confidence interval; filled curve, sampling error distribution. Lower left: metabolite concentrations and E/I ratio (mean +/- SEM). Lower right: null distribution of the group differences generated by permuting subject labels, purple dot: true group difference. **b** No significant group difference was observed in the concentration of Glx (ATX – PLC (n=20:18): permutation test p=0.267). **c** No significant group difference was observed in the concentration of GABA+ (ATX – PLC (n=20:18): permutation test p=0.269). **b** No significant group difference was observed in E/I ratio, defined as the ratio of Glx:GABA+ (ATX – PLC (n=20:18): permutation test p=0.233). **e-f** ROIs used to determine tSNR and small volume correction. **e** 10mm radius sphere centered on the group-average MRS voxel positioned in LOC (Fig. 3c). **f** 10mm radius sphere centered on the group-average MRS voxel positioned in V1. **g** The temporal SNR (tSNR) calculated over the duration of the fMRI acquisition was significantly higher in LOC compared to V1 (LOC – V1: permutation test p<0.001). Upper: DABEST plots. Black dot, mean; black ticks, 95% confidence interval; filled curve, sampling error distribution. Lower right: grey: null distribution of the tSNR differences; purple dot: true regional tSNR difference.

**Supplementary Figure 7 | Relationship between physiological measures of elevated noradrenaline and behavioural measures of overgeneralisation** 

In the ATX group, a single dose of atomoxetine induced expected changes in physiological markers of increased noradrenaline (namely increased pupil response and reduced cortical inhibition, Fig. 3b,e). These physiological markers provide a precise predictor for neural (Fig. 6e-f) and, in turn, behavioural measures of overgeneralisation (Fig. 6a-d). By assessing the relationship between physiological measures of elevated noradrenaline and two behavioural measures of overgeneralisation (namely, *Overgeneralisation errors* and *Mean rank proximity*, Fig. 2f-g respectively, see *Methods*), here we show that a measure of the neural response to atomoxetine, and not mere physiological response to the drug, is necessary to predict overgeneralisation errors in behaviour. **a** No significant relationship was observed between GABA+ in LOC (Fig. 3e) and *Overgeneralisation errors* (Fig. 2f) in either the ATX or PLC group (ATX: n=19, Spearman correlation: r_17_ =-0.135, p=0.581; PLC: n=18, Spearman correlation: r_16_ =-0.267, p=0.282). **b** No significant relationship was observed between GABA+ in LOC (Fig. 3e) and *Mean rank proximity* (Fig. 2g) in either the ATX or PLC group (ATX: n=19, Spearman correlation: r_17_ =0.158, p=0.517; PLC: n=18, Spearman correlation: r_16_ =-0.131, p=0.603). **c** No significant relationship was observed between pupil dilation response (Fig. 3b) and *Overgeneralisation errors* (Fig. 2f) in either the ATX or PLC group (ATX: n=17, Spearman correlation: r_15_ =0.277, p=0.281; PLC: n=17, Spearman correlation: r_15_ =-0.034, p=0.898). **d** No significant relationship was observed between pupil dilation response (Fig. 3b) and *Mean rank proximity* (Fig. 2g) in either the ATX or PLC group (ATX: n=17, Spearman correlation: r_15_=-0.282, p=0.272; PLC: n=17, Spearman correlation: r_15_ =0.232, p=0.371). 

**Supplementary Figure 8 | Additional features of the neural network model** 

Additional features of the neural network model under three different conditions: pre-learning (a), placebo post-learning (b) and atomoxetine post-learning (c). Shown are the average firing rate responses to stimulation of assembly ‘*1*’, together with the distribution of synaptic weights**.** Left panel: Proxy generated by the neural network for XSS contrast used to assess representational overlap with fMRI (Fig. 5b). Each plot shows the difference in average firing rate over the 3.3 s ‘stimulus’ period for assembly ‘*1*’ versus the 3.3 s stimulus period for another assembly (‘*2*’, ‘*3*’, ‘*5*’ or ‘*6*’), relative to the average firing rate over the 3.3 s stimulus period for assembly ‘*1*’ versus assembly ‘*4*’ (the most distal assembly from ‘*1*’). For example, for ‘*12*’ we show: [(average of ‘*1*’ – average of ‘*4*’) - (average of ‘*1*’ – average of ‘*2*’)]. Rightward panels: Distributions of weights of excitatory synapses between assembly ‘*1’* and all other assemblies, 1 second before (grey) and 1 second after (coloured) the 3.3 s ‘stimulus’ period. Arrows indicate the mean value of each distribution. **a-b** In the pre-learning condition and in the post-learning placebo condition, the activities of all assemblies are uniform with respect to activity in *‘4’,* due to the lack of associations between assemblies (a) and sufficient inhibitory rebalancing (b). **c** Under atomoxetine, a gradient can be observed in the activities of assemblies with respect to activity in ‘*4*’, analogous to the XSS effect observed in fMRI data (Fig. 5b).

**Supplementary Figure 9 | Exploring alternative mechanisms in the Placebo network model for generating a spread of association in the underlying memory map: effect of post-learning disinhibition on graded co-activation**

Snapshots of the recurrent spiking neural network. Left: Schematic showing the architecture and parameter conditions of the network. Six cell assemblies are pictured as coloured squares. Excitatory and inhibitory connections are drawn in brown and grey, respectively. Dotted lines indicate weaker connections. Middle: Average firing rate of all excitatory neurons in each assembly, in response to activation of assembly ‘*1*’ via externally driven input (“stimulus”), in the Placebo network. Right: Average firing rates of the excitatory neurons in each assembly during the “stimulus” period. **a** Local disinhibition in the post-learning ‘placebo’ network. Local disinhibition is implemented by downregulating intra-assembly inhibitory to excitatory (IE) connections. Activation of cell assembly ‘*1*’ leads to a graded co-activation across neurons in the other assemblies, relative to their respective distances from assembly *‘1*’. While the average firing rates during the stimulus period (right-hand plot) indicate that the qualitative shape of the graded co-activation matches that shown in the ‘atomoxetine’ network shown in Fig. 4c (where graded co-activation is observed without local disinhibition), co-activity in the post-learning ‘placebo’ network with local disinhibition is of lower magnitude relative to the post-learning ‘atomoxetine’ network without local disinhibition (‘atomoxetine’: dotted pink; ‘placebo’: dotted blue; without local disinhibition). The associations between nodes weren’t learnt under reduced inhibitory firing in the former case. **b** Global disinhibition in the post-learning ‘placebo’ network. Global disinhibition is implemented by decreasing inhibitory firing across the network by downregulating all excitatory to inhibitory (EI) weights to 97%. Activation of cell assembly ‘*1*’ does not result in a pronounced graded co-activation across neurons in the other assemblies, relative to their respective distances from assembly *‘1*’. When EI weights were further decreased to ≤95%, all assemblies were equally co-activated with surplus excitation in the network. 

**Supplementary Figure 10 | Exploring alternative mechanisms in the Placebo network model for generating a spread of association in the underlying memory map:** **effect of assembly overlap on graded co-activation**

Middle (a)/Left (d–e): Average firing rate of all excitatory neurons in each assembly, in response to activation of assembly ‘*1*’ via externally driven input (stimulus). Right (a,d–e): Average firing rates of the excitatory neurons in each assembly. **a** Left: Baseline structural and functional overlap between assemblies immediately after embedding, before they are stabilised by intra-nodal inhibitory rebalancing. Note: grey intra-nodal inhibitory connections are absent. Due to uniform 5% connective sparsity across the network, each assembly is equally connected to all others and equal baseline co-activity is observed across all assemblies in response to driving activity in *‘1’*. **b-d** Effect of assembly overlap on graded co-activity. In the post-learning placebo network, activity in assembly *‘1’* together with a varying percentage of neurons in assemblies *‘2’* and *‘6’* are transiently driven for the ‘stimulus’ period. Thus, neurons in neighbouring assemblies are partially driven by the same afferent input, allowing for both functional and structural overlap. We note that assembly overlap can also be achieved using lateral connections or by allocating the same neurons to more than one assembly. In an experimental setting, these different definitions of ‘assembly overlap’ are not necessarily separable. **b-c** Average excitatory current (b) and average firing rate (c) of all excitatory neurons in assemblies ‘*3*’ and ‘*5*’ (i.e. assemblies that are not directly driven), when varying the percentage overlap between assembly *‘1’* vs. *‘2’* and *‘6’*. Co-activity in assemblies *‘3’* and *‘5’* increases steadily when percentage assembly overlap is ≥40% and a larger percentage of neurons are co-driven in assemblies *‘2’* and *‘6’*. Minimal co-activity is observed when percentage overlap is <40%. The inflection point of 40% is in part determined by the network size, where a lower percentage would be expected in a larger network. **d-e** Snapshots of the network activity of all excitatory neurons when percentage assembly overlap between assembly *‘1’* and assemblies *‘2’* and *‘6’* is either 40% (d) or 80% (e). During the ‘stimulus’ period, co-activity can be observed in assemblies *‘3’* and *‘5’*, demonstrating a spread of association across the network which is more pronounced in **e** with greater assembly overlap.

**Supplementary Figure 11 | Neural network activity showing the effect of heterosynaptic plasticity in stabilising the network via codependent plasticity rules**

Our neural network model employs a "codependent" plasticity rule^47^ which includes both LTP and LTD components. While the total excitation (“E”) to a synapse controls Hebbian LTP, without heterosynaptic plasticity this homosynaptic Hebbian LTP allows excitatory synaptic weights to constantly increase. **a-b** Two snapshots of network behaviour, with (a) and without (b) heterosynaptic plasticity during the initial burn-in stabilisation period of the network, before assemblies are embedded. Network activity is driven only by background activity as described in the *Network and Simulation* section of the *Methods*. Top: Average firing rates through time of six random groups of excitatory neurons, where each group is composed of 100 neurons. Middle: Synaptic weights through time of 500 randomly selected excitatory synapses (w_EE) from the network. Bottom: Average net excitatory current (implemented in eq. 5, eq. 6, eq. 8 of Methods), shown through time for 100 randomly selected excitatory neurons in the network. **a** With heterosynaptic plasticity, the network stabilises after ~20 seconds of background activity with reasonable firing rates, excitatory weights and excitatory currents, thus establishing a balanced recurrent spiking neural network. **b** Without heterosynaptic plasticity, the excitatory-to-excitatory weights (w_EE) are not sufficiently stabilised. As a consequence, w_EE reach their maximum possible value (1nS), leading to increasing exponential excitatory currents in the network, and, in turn, implausible excitatory firing rates are attained in the network.

**Supplementary Figure 12 | Group effects for the neural spread of association effect**

**a** Anatomical ROI of parahippocampus-hippocampus used for small volume correction.  **b-d** T-statistic maps showing the neural spread of association effect.  **b** Spread of association quantified using the second GLM, with the full 11-node task structure used to derive the parametric regressor. In the PLC group, no significant neural spread of association was observed in parahippocampus-hippocampus (SVC with parahippocampus-hippocampus ROI, t_20_=3.85, p=0.122, MNI coordinates). **c-d** Spread of association quantified using the third GLM, with the 6-node output from the neural network model. **c** In the ATX group, no significant effect was observed for the neural spread of association in parahippocampus-hippocampus (SVC with parahippocampus-hippocampus ROI, n=22, t_21_=3.86, p=0.115, MNI coordinates, Supplementary Table 4), although a post hoc test revealed a significant effect in right hippocampus (SVC with right hippocampus ROI, n=22, t_21_=3.86, p=0.041, Fig. 5g). **d** In the PLC group, no significant effect was observed for the neural spread of association in parahippocampus-hippocampus (SVC with parahippocampus-hippocampus ROI, n=21, t_20_=3.06 p=0.424, MNI coordinates). A post hoc test revealed no significant effect in right hippocampus (SVC with right hippocampus ROI, n=21, t_20_=3.06, p=0.172) 

**Supplementary Figure 13 | Correlations with the neural spread of association effect in the PLC group and correlation group differences**

T-statistic maps showing correlations with the spread of association effect. **a** In the PLC group, no significant relationship was observed between the neural spread of association and ‘Overgeneralisation errors’ reported in behaviour (SVC with parahippocampus-hippocampus ROI, n=21, t_19_=2.32, p=0.825, MNI coordinates). **b** In the PLC group, no significant relationship was observed between the neural spread of association and ‘Mean rank proximity’ reported in behaviour (SVC with parahippocampus-hippocampus ROI, n=21, t_19_=2.23, p=0.858, MNI coordinates). **c** In the PLC group, no significant correlation was observed between the concentration of GABA+ in LOC and the neural spread of association effect (SVC with parahippocampus-hippocampus ROI, n=17, t_14_=3.15, p=0.461). **d** No significant group difference was observed for the relationship between GABA+ in LOC and the neural spread of association effect in hippocampus-parahippocampus (ATX – PLC (n=19:17): SVC with parahippocampus-hippocampus ROI, t_30_=3.17, p=0.195, MNI coordinates). **e** In the PLC group, no significant correlation was observed between the pupil dilation response to surprising stimuli and the neural spread of association effect (SVC with parahippocampus-hippocampus ROI, n=12, t_8_=3.63, p=0.501 MNI coordinates). **f** A trend towards a significant group difference was observed for the relationship between the pupil dilation and the neural spread of association in parahippocampus-hippocampus (ATX – PLC (n=15:12): SVC with parahippocampus-hippocampus ROI, t_19_=3.45, p=0.078, MNI coordinates, Supplementary Table 12). Post hoc tests revealed significant group differences in left and right hippocampus and left parahippocampus (ATX – PLC (n=15:12): SVC with left hippocampus ROI, t_19_=4.13, p=0.026; SVC with right hippocampus ROI, t_19_=3.89, p=0.042; SVC with left parahippocampus ROI, t_19_=3.48, p=0.046).

**Supplementary Figure 14 | Learning time did not differ between groups and did not relate to behavioural, neural or physiological measures in the ATX group**

**a** On Day 1, participants learned the stimulus associations in 29.72 minutes on average. There was no significant difference in learning time between groups (ATX – PLC (n=22:22): 2-sided permutation test p=0.409). Upper: Bootstrap-coupled estimation (DABEST) plots. Black dot, mean; black ticks, 95% confidence interval; filled curve, sampling error distribution. Lower left: memory accuracy (mean +/- SEM). Lower right: null distribution of the group differences generated by permuting subject labels, purple dot: true group difference. **b** In the ATX group, no relationship was observed between learning time and overgeneralisation errors (Fig. 2f) (ATX: n=22, Spearman correlation: r_20_=0.038, p=0.865). In the PLC group, learning time positively predicted overgeneralisation errors (PLC: n=22, Spearman correlation: r_20_ =0.525, p=0.013). **c** No relationship was observed between learning time and mean error proximity rank (Fig. 2g) in either the ATX or PLC group (ATX: n=22, Spearman correlation: r_20_=0.024, p=0.916; PLC: n=22, Spearman correlation: r_20_ =-0.366, p=0.094). **d** No relationship was observed between learning time and the XSS regressor for neural spread of association (Fig. 5c, Supplementary Fig. 12b) in either the ATX or PLC group (ATX: n=22, Spearman correlation: r_20_=-0.321, p=0.156; PLC: n=21, Spearman correlation: r_19_=-0.368, p=[0.](http://0.xxx/)102). **e** In the ATX group, no relationship was observed between learning time and GABA+ in LOC (Fig. 3e) (ATX: n=19, Spearman correlation: r_17_=0.270, p=0.262). In the PLC group, learning time positively predicted GABA+ in LOC (PLC: n=18, Spearman correlation: r_16_ =-0.501, p=0.036). **f** No relationship was observed between learning time and pupil dilation response (Fig. 3b) in either the ATX or PLC group (ATX: n=17, Spearman correlation: r_15_=0.029, p=0.913; PLC: n=17, Spearman correlation: r_15_ =0.069, p=0.795).

**Supplementary Tables**

**Supplementary Table 1 | Effectiveness of blinding procedure** 

Effectiveness of the blinding procedure assessed using Bang’s blinding index^75^ (BI) (see *Methods*). Importantly, the BI for ATX indicates successful blinding in this group, for both the participants and experimenter. The BI for the PLC of >0.2 for both participants and experimenter, indicating a tendency to correctly guess that PLC participants received placebo. 

| **Participants** |  |  |  | **Experimenter** |  |  |  |
| --- | --- | --- | --- | --- | --- | --- | --- |
| Condition | BI | Confidence interval |  | Condition | BI | Confidence interval |  |
|  |  | Lower bound | Upper bound |  |  | Lower bound | Upper bound |
| ATX | -0.18 | -0.59 | 0.23 | ATX | 0.00 | -0.42 | 0.42 |
| PLC | 0.36 | -0.03 | 0.75 | PLC | 0.64 | 0.31 | 0.96 |

**Supplementary Table 2 | Across all participants, repetition suppression as an index for the neural representation of the task stimuli; related to Supplementary Figure 5.** 

Brain regions showing significant repetition suppression for the task stimuli, as described in Supplementary Fig. 5b. No brain regions survived FWE whole-brain correction at the cluster-level (p<0.05). Statistics reported in LOC (Supplementary Fig. 5a) are peak-level FWE corrected using a small-volume correction method.

| Brain region | P _FWE-corr_ | T _peak level_ | Coordinate |  |  |
| --- | --- | --- | --- | --- | --- |
|  |  |  | **x** | **y** | **z** |
| LOC | P=0.010 | 4.23 | -40 | -55 | -8 |

**Supplementary Table 3 | In the ATX group, cross-stimulus suppression as an index for spread of association**; related to Fig. 5. 

Brain regions showing significant cross-stimulus suppression for the parametric spread of association effect, as described in Fig. 5b. No brain regions survived FWE whole-brain correction at the cluster-level (p<0.05). Statistics reported in the parahippocampus-hippocampus (Supplementary Fig. 12a) are peak-level FWE corrected using a small-volume correction method. Post-hoc tests in parahippocampus and hippocampus are peak-level FWE corrected using a small-volume correction method, with significant results reported (Fig. 5c,d).

| Brain region | P _FWE-corr_ | T _peak level_ | Coordinate |  |  |
| --- | --- | --- | --- | --- | --- |
|  |  |  | **x** | **y** | **z** |
| Parahippocampus & hippocampus | P=0.032 | 4.56 | 22 | -36 | -18 |
| Parahippocampus & hippocampus | P=0.048 | 4.35 | 28 | -13 | -16 |
| Right parahippocampus | P=0.005 | 4.56 | 22 | -36 | -18 |
| Right hippocampus | P=0.017 | 4.35 | 28 | -13 | -16 |
| Left parahippocampus | P=0.043 | 3.51 | -21 | -42 | -13 |
| Left hippocampus | P=0.354 | 2.48 | -26 | -38 | 0 |

**Supplementary Table 4 | In the ATX group, cross-stimulus suppression as an index for spread of association using neural network model output**; related to Fig. 5. 

Brain regions showing significant cross-stimulus suppression for the parametric spread of association effect, as described in Fig. 5b. No brain regions survived FWE whole-brain correction at the cluster-level (p<0.05). Statistics reported in the parahippocampus-hippocampus (Supplementary Fig. 12a) are peak-level FWE corrected using a small-volume correction method. 

| Brain region | P _FWE-corr_ | T _peak level_ | Coordinate |  |  |
| --- | --- | --- | --- | --- | --- |
|  |  |  | **x** | **y** | **z** |
| Parahippocampus & hippocampus | P=0.115 | 3.86 | 26 | -16 | -18 |
| Parahippocampus & hippocampus | P=0.191 | 3.56 | -22 | -42 | -13 |

**Supplementary Table 5 | Group difference (ATX vs. PLC) for cross-stimulus suppression as an index for spread of association using neural network model output;** related to Fig. 5. 

Brain regions showing a significant group difference (ATX – PLC) in correlation between cross-stimulus suppression as an index for the spread of association (as described in Fig. 5b) and overgeneralisation errors reported in behaviour. No brain regions survived FWE whole-brain correction at the cluster-level (p<0.05). Statistics reported in the parahippocampus-hippocampus Supplementary Fig. 12a) are peak-level FWE corrected using a small-volume correction method.

| Brain region | P _FWE-corr_ | T _peak level_ | Coordinate |  |  |
| --- | --- | --- | --- | --- | --- |
|  |  |  | **x** | **y** | **z** |
| Parahippocampus & hippocampus | P=0.347 | 2.96 | 27 | -19 | -18 |
| Parahippocampus & hippocampus | P=0.436 | 2.82 | 15 | -10 | - |

**Supplementary Table 6 | In the ATX group, correlation between cross-stimulus suppression as an index for spread of association and overgeneralisation errors in behaviour;** related to Fig. 6. 

Brain regions showing significant correlation between cross-stimulus suppression for the spread of association effect (as described in Fig. 5b) and the behavioural measure of overgeneralisation errors (Fig. 2f). No brain regions survived FWE whole-brain correction at the cluster-level (p<0.05). Statistics reported in the parahippocampus-hippocampus (Supplementary Fig. 12a) are peak-level FWE corrected using a small-volume correction method. Post hoc tests in hippocampus and parahippocampus are peak-level FWE corrected using a small-volume correction method, with significant results reported (Fig. 6a). 

| Brain region | P _FWE-corr_ | T _peak level_ | Coordinate |  |  |
| --- | --- | --- | --- | --- | --- |
|  |  |  | **x** | **y** | **z** |
| Parahippocampus & hippocampus | P=0.002 | 6.16 | 34 | -24 | -12 |
| Right hippocampus | P=0.001 | 6.16 | 34 | -24 | -12 |
| Right parahippocampus | P=0.131 | 2.90 | 22 | -43 | -12 |
| Left hippocampus | P=0.148 | 3.16 | -14 | -38 | -1 |
| Left parahippocampus | p=0.365 | 2.18 | -14 | -42 | -6 |

**Supplementary Table 7 | In the ATX group, correlation between cross-stimulus suppression as an index for spread of association and mean rank proximity in behaviour;** related to Fig. 6. 

Brain regions showing significant correlation between cross-stimulus suppression for the spread of association effect (Fig. 5b) and the behavioural measure of error proximity rank (Fig. 2g). A whole brain effect was observed in premotor cortex. Statistics reported in the parahippocampus-hippocampus (Supplementary Fig. 12a) are peak-level FWE corrected using a small-volume correction method. Post hoc tests in hippocampus and parahippocampus are peak-level FWE corrected using a small-volume correction method, with significant results reported (Fig. 6b). 

| Brain region | P _FWE-corr_ | T _peak level_ | Coordinate |  |  |
| --- | --- | --- | --- | --- | --- |
|  |  |  | **x** | **y** | **z** |
| Premotor cortex | P=0.003 | 8.62 | -21 | -13 | 42 |
| Parahippocampus & hippocampus | P=0.003 | 5.92 | 32 | -22 | -12 |
| Right hippocampus | P=0.001 | 5.92 | 32 | -22 | -12 |
| Left hippocampus | P=0.346 | 2.54 | -33 | -22 | -14 |
| Right parahippocampus | P=0.403 | 2.05 | 28 | -43 | -8 |
| Left parahippocampus | P=0.613 | 1.58 | -28 | -28 | -19 |

**Supplementary Table 8 | Group difference (ATX vs. PLC) for the correlation between cross-stimulus suppression as an index for spread of association and the behavioural measure of overgeneralisation errors;** related to Fig. 6. 

Brain regions showing a significant group difference (ATX – PLC) in correlation between cross-stimulus suppression as an index for the spread of association (Fig. 5b) and overgeneralisation errors reported in behaviour (Fig. 2F). No brain regions survived FWE whole-brain correction at the cluster-level (p<0.05). Statistics reported in the parahippocampus-hippocampus are peak-level FWE corrected using a small-volume correction method. Post hoc tests in hippocampus and parahippocampus (Supplementary Fig. 12a) are peak-level FWE corrected using a small-volume correction method, with significant results reported (Fig. 6c).

| Brain region | P _FWE-corr_ | T _peak level_ | Coordinate |  |  |
| --- | --- | --- | --- | --- | --- |
|  |  |  | **x** | **y** | **z** |
| Parahippocampus & hippocampus | P=0.011 | 4.51 | 36 | -22 | -14 |
| Right hippocampus | P=0.004 | 4.51 | 36 | -22 | -14 |
| Left hippocampus | P=0.392 | 2.27 | -15 | -40 | 0 |
| Left parahippocampus | P=0.575 | 1.59 | -28 | -28 | -20 |
| Right parahippocampus | P=0.576 | 1.52 | 36 | -28 | -18 |

**Supplementary Table 9 | Group difference (ATX vs. PLC) for the correlation between cross-stimulus suppression as an index for spread of association and mean rank proximity in behaviour;** related to Fig. 6. 

Brain regions showing a significant group difference (ATX – PLC) in correlation between cross-stimulus suppression as an index for the spread of association (Fig. 5b) and mean rank proximity reported in behaviour (Fig. 2g). No brain regions survived FWE whole-brain correction at the cluster-level (p<0.05). Statistics reported in the parahippocampus-hippocampus are peak-level FWE corrected using a small-volume correction method. Post hoc tests in hippocampus and parahippocampus (Supplementary Fig. 12a) are peak-level FWE corrected using a small-volume correction method, with significant results reported (Fig. 6d).

| Brain region | P _FWE-corr_ | T _peak level_ | Coordinate |  |  |
| --- | --- | --- | --- | --- | --- |
|  |  |  | **x** | **y** | **z** |
| Parahippocampus & hippocampus | P=0.037 | 4.03 | 33 | -24 | -12 |
| Right hippocampus | P=0.013 | 4.03 | 33 | -24 | -12 |
| Left hippocampus | P=0.331 | 2.40 | -33 | -22 | -14 |
| Left parahippocampus | P=0.490 | 1.78 | -15 | -31 | -14 |
| Right parahippocampus | P=0.741 | 0.82 | 36 | -28 | -18 |

**Supplementary Table 10 | In the ATX group, correlation between GABA+ in LOC and cross-stimulus suppression as an index for spread of association;** related to Fig. 6. 

Brain regions showing significant correlation with GABA+ in LOC (Fig. 3e) and cross-stimulus suppression as an index for spread of association in the underlying memory map (Fig. 5b). No brain regions survived FWE whole-brain correction at the cluster-level (p<0.05). Statistics reported in parahippocampus-hippocampus (Supplementary Fig. 12a) are peak-level FWE corrected using a small-volume correction method. Post hoc tests in hippocampus and parahippocampus are peak-level FWE corrected using a small-volume correction method, with significant results reported (Fig. 6e). 

| Brain region | P _FWE-corr_ | T _peak level_ | Coordinate |  |  |
| --- | --- | --- | --- | --- | --- |
|  |  |  | **x** | **y** | **z** |
| Parahippocampus & hippocampus | P=0.047 | 4.70 | -21 | -44 | -10 |
| Left Parahippocampus | P=0.008 | 4.70 | -21 | -44 | -10 |
| Right Parahippocampus | P=0.023 | 4.07 | 27 | -37 | -14 |
| Left hippocampus | P=0.196 | 3.06 | -30 | -37 | -7 |
| Right hippocampus | P=0.645 | 1.98 | 36 | -18 | -19 |

**Supplementary Table 11 | In the ATX group, correlation between pupil dilation response to surprising stimuli and cross-stimulus suppression as an index for spread of association;** related to Fig. 6. 

Brain regions showing significant correlation between the pupil dilation response to surprising stimuli (Fig. 3b) and cross-stimulus suppression as an index for the spread of association effect (Fig. 5b). No brain regions survived FWE whole-brain correction at the cluster-level (p<0.05). Statistics reported in the parahippocampus-hippocampus (Supplementary Fig. 12a) are peak-level FWE corrected using a small-volume correction method. Post hoc tests in hippocampus and parahippocampus are peak-level FWE corrected using a small-volume correction method, with significant results reported (Fig. 6f). 

| Brain region | P _FWE-corr_ | T _peak level_ | Coordinate |  |  |
| --- | --- | --- | --- | --- | --- |
|  |  |  | **x** | **y** | **z** |
| Parahippocampus & hippocampus | P=0.066 | 5.15 | 21 | -18 | -16 |
| Right hippocampus | P=0.023 | 5.15 | 21 | -18 | -16 |
| Left hippocampus | P=0.066 | 4.32 | -22 | -16 | -19 |
| Left parahippocampus | P=0.095 | 3.48 | -12 | -42 | -7 |
| Right parahippocampus | P=0.222 | 2.73 | 30 | -38 | -12 |

**Supplementary Table 12 | Group difference (ATX vs. PLC) for the correlation between cross-stimulus suppression as an index for spread of association and the pupil dilation response to surprising stimuli;** related to Supplementary Fig. 13. 

Brain regions showing a trend group difference (ATX – PLC) in correlation between the pupil dilation response to surprising stimuli (Fig. 3b) and cross-stimulus suppression as an index for the spread of association effect (Fig. 5b). No brain regions survived FWE whole-brain correction at the cluster-level (p<0.05). Statistics reported in the parahippocampus-hippocampus (Supplementary Fig. 12a) are peak-level FWE corrected using a small-volume correction method. Post hoc tests in hippocampus and parahippocampus are peak-level FWE corrected using a small-volume correction method, with significant results reported (Supplementary Fig. 13f).

| Brain region | P _FWE-corr_ | T _peak level_ | Coordinate |  |  |
| --- | --- | --- | --- | --- | --- |
|  |  |  | **x** | **y** | **z** |
| Parahippocampus & hippocampus | P=0.078 | 4.13 | -27 | -24 | -19 |
| Left hippocampus | P=0.026 | 4.13 | -27 | -24 | -19 |
| Right hippocampus | P=0.042 | 3.89 | 24 | -26 | -12 |
| Left parahippocampus | P=0.046 | 3.48 | -21 | -28 | -22 |
| Right parahippocampus | P=0.088 | 3.09 | 15 | -34 | -13 |
